# Preface to the First Volume of the Medico-Chirurgical Review. (Analytical Series.)

**Published:** 1820-06-01

**Authors:** 


### PREFACE

TO THE

FIRST VOLUME

OF

### THE MEDICO-CHIRURGICAL REVIEW.

(Analytical Series.)

*On closing the First Volume of our Analytical Series, it is a paramount duty to return unfeigned thanks to Subscribers and the Profession at large, for a degree of countenance and encouragement which we could not have expected, and probably did not deserve. We may safely affirm, however, that we have endeavoured to merit this patronage—and we hope it is evident that, with time and experience, the Journal has progressively improved. Often, indeed, when reflecting on the arduous nature of our undertaking, we have shuddered at the danger of the attempt. But the great difficulties are conquered—exuberance of assistance is perpetually at hand—and what was once an anxious task, is now a mental recreation.*

*The organization of the Journal has grown out of the intellectual state of the age in which we live, and the swelling stream of general as well as professional literature. Every one, who is not interested in distorting truth, will now admit, that an* ANALYTICAL REVIEW, *on a large scale, is necessary, not only to reflect the great features of medical science diffusively through the profession, but to fix them in a form of record conservative of what is most valuable, and calculated alike for present* perusal *and subsequent* reference. *The* latter *object has been, and will ever be, kept steadily in view, from a thorough conviction that, by declining all temporary matter, and registering that only, which will be read with interest, and referred to with advantage, we are constructing a work, the volumes of which will become more valuable in proportion to their age. What an inestimable treasure would now be an Analytical Review of all that has been published during the last fifty years!—And with what interest will a future generation peruse these concentrated records of the medical science of the present æra, distinguished as it is, by intensity of thought, accuracy of observation, rigour of deduction, intrepidity of experiment, fertility of resource, and emancipation from prejudice!*

*In medical society there are necessarily gradations of rank, and varieties of character. There are not a few, whose toilsome professional avocations leave no time for extensive reading—there are many whose "res angusta domi" confines the library within narrow limits—and there is a large portion scattered over the globe,* per mare ac terram, *who have no possible means of consulting the original works which daily issue from the press. Yet, in these different classes, no man can hope to retain his station in the estimation of his brethren, keep pace with the progress of science, or do justice to the art which he professes, without, at least, a periodical work to inform him what others are doing, and what others are thinking in the world around him.*

*As one of the now numerous candidates for performing this office, the Medico-Chirurgical Review has presented itself before the profession, in its present form, without any wish to depreciate its cotemporaries, or to magnify the necessity that existed for new labourers in the vineyard of periodical literature. Such an attempt would be equally impolitic and illiberal. The* PUBLIC *is a stern, inflexible, and, finally, unerring tribunal, before which it is unnecessary to plead the cause of merit, and useless to varnish infirmity. Friends may flatter, and enemies may defame, but the Public at large do justice, because they are far removed from the sphere of personal feeling and influence. It is to this tribunal—and to this tribunal* alone, *that we shall ever appeal.*

*There is, however, a class of medical society, numerically not great, but potentially important, on which this Journal may, probably, have some claims—it is the class of* MEDICAL AUTHORS. *To analytically pourtray their works before the whole professional circle, leaving the judgment in general, to the Public itself, is no mean service to authors—no common advantage to readers—no sinecure office for reviewers—no inconsiderable check on hypercritics. Whether the* MEDICO-CHIRURGICAL REVIEW *executes this task, with any degree of credit to itself, or satisfaction to the Public, it is for that Public to decide. If it does—their patronage will, in justice, be continued—if it does not, it will, with equal justice, be withdrawn. Literary competitions, in their effects, are directly the reverse of political struggles for ascendancy. Success may elevate, or failure depress individuals; but the contest is uniformly beneficial to the community at large. Whoever wears the laurel, the Public gains the prize.*

